# Clonal distribution of *BCR-ABL1* mutations and splice isoforms by single-molecule long-read RNA sequencing

**DOI:** 10.1186/s12885-015-1046-y

**Published:** 2015-02-12

**Authors:** Lucia Cavelier, Adam Ameur, Susana Häggqvist, Ida Höijer, Nicola Cahill, Ulla Olsson-Strömberg, Monica Hermanson

**Affiliations:** 1 Department of Immunology, Genetics and Pathology, Science for Life Laboratory, Uppsala University, Uppsala, Sweden; 2 Department of Medical Sciences, Haematology, Uppsala University, Uppsala, Sweden

## Abstract

**Background:**

The evolution of mutations in the *BCR-ABL1* fusion gene transcript renders CML patients resistant to tyrosine kinase inhibitor (TKI) based therapy. Thus screening for *BCR-ABL1* mutations is recommended particularly in patients experiencing poor response to treatment. Herein we describe a novel approach for the detection and surveillance of *BCR-ABL1* mutations in CML patients.

**Methods:**

To detect mutations in the *BCR-ABL1* transcript we developed an assay based on the Pacific Biosciences (PacBio) sequencing technology, which allows for single-molecule long-read sequencing of *BCR-ABL1* fusion transcript molecules. Samples from six patients with poor response to therapy were analyzed both at diagnosis and follow-up. cDNA was generated from total RNA and a 1,6 kb fragment encompassing the *BCR-ABL1* transcript was amplified using long range PCR. To estimate the sensitivity of the assay, a serial dilution experiment was performed.

**Results:**

Over 10,000 full-length *BCR-ABL1* sequences were obtained for all samples studied. Through the serial dilution analysis, mutations in CML patient samples could be detected down to a level of at least 1%. Notably, the assay was determined to be sufficiently sensitive even in patients harboring a low abundance of *BCR-ABL1* levels. The PacBio sequencing successfully identified all mutations seen by standard methods. Importantly, we identified several mutations that escaped detection by the clinical routine analysis. Resistance mutations were found in all but one of the patients. Due to the long reads afforded by PacBio sequencing, compound mutations present in the same molecule were readily distinguished from independent alterations arising in different molecules. Moreover, several transcript isoforms of the *BCR-ABL1* transcript were identified in two of the CML patients. Finally, our assay allowed for a quick turn around time allowing samples to be reported upon within 2 days.

**Conclusions:**

In summary the PacBio sequencing assay can be applied to detect *BCR-ABL1* resistance mutations in both diagnostic and follow-up CML patient samples using a simple protocol applicable to routine diagnosis. The method besides its sensitivity, gives a complete view of the clonal distribution of mutations, which is of importance when making therapy decisions.

## Background

Treatment of chronic myeloid leukemia (CML) has advanced with the introduction of tyrosine kinase inhibitors (TKI) that target the *BCR-ABL1* fusion protein such as imatinib, and furthermore with second line inhibitors such as dasatinib, nilotinib, bosutinib and ponatinib. To measure the effect of TKI therapy, real-time quantitative PCR (RQ-PCR) of the *BCR-ABL1* fusion transcript is routinely performed and transcript levels are followed longitudinally for each patient. However, in case of limited TKI response or of progression to accelerated phase or blast crisis, mutational analysis of the ABL1 kinase domain should be performed, as stated by the ELN (European Leukemia Net) recommendations [1], since evolution of such mutations may lead to poor response to TKIs. One mutation of particular importance for clinical investigations is the multi-resistant substitution T315I, resulting in an amino acid change within the p-loop binding site. Furthermore, rare mutations within the regulatory domain of *ABL1* have also been reported to lead to TKI resistance in patients without kinase domain mutations [2]. A further concern is the presence of concurrent *BCR-ABL1* mutations, which may also hamper successful therapy [3-5]. Ideally, mutations in both regulatory and kinase domains as well as co-existing mutations should therefore be detected as early as possible, prior to an expansion of resistant clones. In addition to point mutations, the *BCR-ABL1* protein can be affected by alterations in splicing where whole exons, or smaller parts of exons, are included or skipped from the main transcript [6,7]. The clinical significance of splice isoforms remains to be elucidated, mainly because their detection has until recently required time consuming cloning steps prior to sequencing.

Today, various assays including Sanger sequencing and quantitative RT-PCR are routinely applied for *BCR-ABL1* mutation detection. While Sanger sequencing has limited sensitivity, real time reverse transcription PCR requires mutation specific panels with separate standard curves and variable sensitivity. A further limitation is that these assays can typically not resolve the patterns of co-existing mutations. With the introduction of massively parallel sequencing (MPS) technologies it is now possible to study these mutations at an entirely new level of resolution. In recent studies performed on the Roche 454 system, *BCR-ABL1* mutations were detected at a higher sensitivity as compared to Sanger sequencing [8,9]. However, although the 454 system produces longer sequences than most other instruments, these still cannot span the complete transcript. Thus, MPS studies have until now mainly been based on sequencing of smaller fragments of *BCR-ABL1*, often amplified in two successive rounds using a nested PCR approach. This strategy not only limits the analysis to a portion of the transcript but it is likely to introduce a bias in the resulting mutation frequencies.

Here we present for the first time an assay to directly investigate the entire 1,578 bp *BCR-ABL1* major fusion transcript, amplified from a single PCR reaction and sequencing on the Pacific Biosciences (PacBio) RSII system. When comparing available MPS platforms, the PacBio instrument is particularly attractive for *BCR-ABL1* analysis. In addition to enabling a rapid workflow at a relatively low cost, the PacBio system produces reads sufficiently long to span across a full length *BCR-ABL1* molecule. This allows for an immediate detection of compound mutations and splice isoforms.

## Methods

### Patient samples

Six patients diagnosed with CML at Uppsala University Hospital, all receiving imatinib as first line treatment were included in this study. All six patients showed limited or no molecular response to TKI treatment. Two of the patients were included in a ponatinib study (PACE, study nr AP24534-10-201, phase 2 clinical trial, Ariad Pharmaceuticals, MA, USA). Samples at diagnosis, and following TKI therapy were tested. For a complete list of all patient samples sequenced in this study, see Table 1. The clinical characteristics of each patient are given in further detail in the results section.

**Table 1:** **Characteristics of the patient samples included in this study**

	Age/sex	Sokal score	Karyotype	Diagnosis	Time after diag (m)	CCS reads	PacBio results	Sanger results
**Patient 1**	43/M	high risk	46,XY,t(9;22)(q34;q11)[20]	CP CML	0	37432	wildtype	ND
			ND	CP CML	7	32703	T315I (3.9%)	wildtype
			ND	CP CML	9	30251	T315I (53,5%)	ND
**Patient 2**	70/M	high risk	46,XY,t(9;22)(q34;q11)[25]	CP CML	0	23089	wildtype	ND
			46,XY,t(9;22)(q34;q11)[19]/		6	27633	wildtype	ND
			46,XY,t(9;22)(q34;q11)[4]/46,XY,idem,del(11)(q14)[16]	AP CML	43	34467	wildtype	wildtype
			ND but later sample shows 46,XY,t(9;22)(q34;q11)del(11)(q14)[20]		64	41963	T315I (98%)	T315I
**Patient 3**	65/M	high risk	46,XY,t(9;22)(q34;q11)[25]	CP CML	0	35377	wildtype	ND
			46,XY,t(9;22)(q34;q11)[24]/46,XY[1]	CP CML	3		wildtype	ND
			46,XY,t(9;22)(q34;q11)[6]/47sl,i(17)(q10),add(20)(p13),+mar[4]/46,XY[10]	AP CML	49	39685	T315I (88,9%), F359C (4,2%)	T315I
			46,XY,t(9;22)(q34;q11)[3]/46,XY,del(5q)[6]/46,XY[10]	AP CML	55	42642	T315I (94,8%), F359C (2,2%), D276G (1,8%), H396R (1%)	T315I
**Patient 4**	61/F	high risk	46,XX,t(9;22)(q34;q11)[25]	CP AML	0	36658	wildtype	ND
			46,XX[20]	CP CML	111	41922	F359I (83,1%), T315I (13,5%)	F359I, T315I
**Patient 5**	66/M		ND	CP CML	0	24062	wildtype	ND
			46,XY,del(6)(q2?1;q2?3),-7,t(9;22)(q34;q11)[20]	Blast crisis	4	28446	Y253H (94,8%), E255V (1,8%)	Y253H
**Patient 6**	65/M		46,XY,t(9;22)(q34;q11)[20]	CP CML	0	32982	wildtype	ND
			46,XY,t(9;22)(q34;q11)[11]/	CP CML	7	29221	4 isoforms	wildtype
			46,XY[20]	CP CML	13	34726	3 isoforms	ND

### Ethics statement

This study was performed in accordance with the Declaration of Helsinki. The Ethical Committee at Uppsala University, Dnr 00–623, approved this study. Written informed consent was obtained from the patients.

### RNA extraction and cDNA synthesis

RNA was extracted from peripheral blood or bone marrow samples using a TRIzol®, (Thermoscientific, MA, USA) standard protocol and quantified by the nanodrop 2000 instrument (Thermoscientific, MA, USA). cDNA was synthesized using the SMARTer™ PCR cDNA synthesis kit (ClonTech, CA, USA), using 1000 ng total RNA.

### Dilution series of BCR-ABL1 samples

Using a quantitative real time reverse transcription PCR assay, total *BCR-ABL1* p210 transcripts were quantified in the 49 month post diagnosis sample for patient three (P3) and a wild type sample at diagnosis. The two samples where then diluted to contain the same amount of *BCR-ABL1* copies/microliter. The P3 sample was then serially diluted into the sample wild type at varying amounts 50%, 10%, 1% and 0.5%.

### Library preparation and PacBio sequencing

Long range PCR amplification of the *BCR-ABL1* p210 transcript was performed using the Clontech Advantage PCR kit (Clontech, CA, USA). Using *BCR-ABL1* specific primers (*BCR* exon 12, forward 5’- tga cca act cgt gtg tga aac tc – 3’ and *ABL1* exon9/10, reverse 5’ - tcc act tcg tct gag ata ctg gat t - 3’) a 1578 bp cDNA amplicon ranging from exon 12 (including e13 and e14 breakpoint variants) in *BCR* to exon 9 in A*BL1* was obtained. The cDNA reaction was diluted to 50 μl using TE buffer. 5 μl of diluted cDNA were used in a 50 μl PCR reaction, following the manufacturers instructions. Samples were placed into a preheated 95°C thermocycler and cycled as follows; initial denaturation for 1 minute at 95°C followed by 30 cycles of 15 seconds at 95°C, 30 seconds at 61°C and 3 minutes at 68°C. Following amplification, amplicon size was confirmed using the bioanalyser 12000 kit (Agilent, CA, USA) and the concentration confirmed by Qubit assay (Life Technologies, CA, USA).

SMRTbell™ libraries were produced using the Pacific Biosciences 1.0 template preparation kit according to the manufacturer’s instructions. SMRTbells™ were constructed and sequenced following the recommended pacific biosciences 2kb template preparation protocol. In brief, cDNA amplicons (300–750 ngs) underwent end-repair and adaptor ligation processes to generate SMRTbell™ libraries for circular consensus sequencing. Libraries were then subjected to exo treatment and Ampure bead wash procedures for clean up. SMRTbell™ libraries were quantified using the Qubit assay and library size was confirmed using the bioanalyser 12000 kit. Following SMRTbell™ construction, v2 primers and P4 polymerase were annealed and enzyme bound complexes attached to magnetic beads for loading. Each SMRTbell™ amplicon library was loaded on to one SMRT cell and sequenced on the PacBio RS II instrument using C2 chemistry and a 120 minute movie time.

### PacBio data analysis and mutation detection

Detection of mutations in the PacBio data was performed using the ‘Minor and Compound Variants’ plug-in available in v2.0.1 of the PacBio SMRT Analysis Portal. Custom R scripts were used to study the mutational composition in patients carrying several mutations. This was done by looping through all circular consensus (CCS) reads and recording the mutational composition in each individual read. For a read to be present in the analysis of compound mutations, 20 bases in a window surrounding each mutation were required to match perfectly to the *BCR-ABL1* p210 transcript reference sequence*.* In this way only reads with relatively high quality were used, thereby reducing the effects of sequencing errors.

### Detection of BCR-ABL1 isoforms

*BCR-ABL1* splice isoforms were identified from full-length CCS reads spanning the length of the entire transcript. For a splice isoform to be reported, we required at least two independent CCS reads to contain identical nucleotide sequences over the entire length of the transcript.

## Results

This study shows the applicability of PacBio sequencing to detect *BCR-ABL1* mutations in CML patients with poor molecular response to treatment. As a proof of concept we have analyzed patient samples previously analyzed by standard routine methods and all but one were positive for TKI-resistance mutations. Besides confirming all previously detected mutations we could detect 5 new mutations. Most importantly, we could determine the clonal distribution of mutations, thereby bypassing the need of cloning experiments. We could establish if mutations were present in the same (compound) or in different molecules (independent), which is clinically relevant for therapy decision-making. Furthermore we could simultaneously detect transcripts isoforms.

### Sequencing and sensitivity of the assay

**Figure 1:**
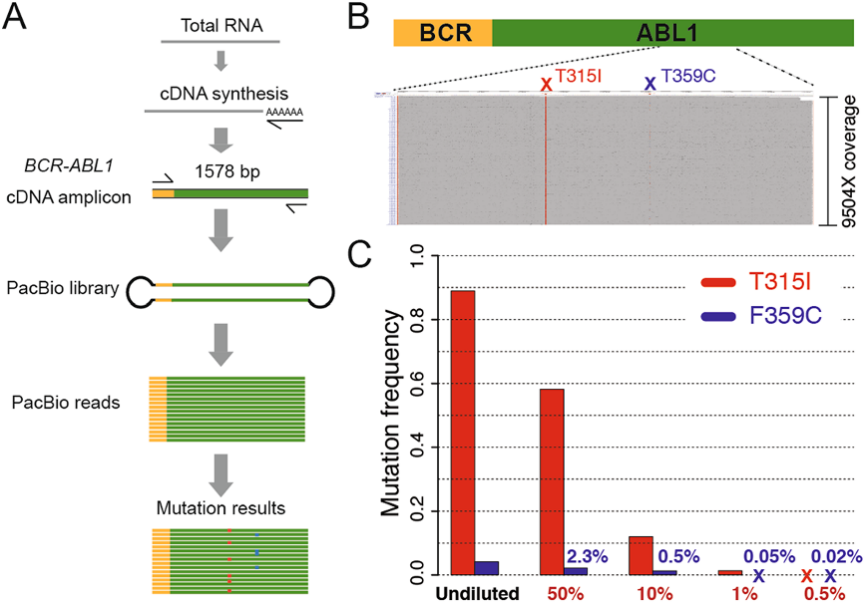
**Overview and evaluation of*BCR-ABL1*mutation detection using the PacBio sequencing. A)** Schematic overview of the workflow. Total RNA was used to generate a 1578 bp long *BCR-ABL1* fusion transcript cDNA amplicon. PacBio adaptors were ligated to the amplicon and the resulting library was sequenced on a PacBio SMRT cell. The data analysis detected *BCR-ABL1* mutations down to a frequency of at least 1%, as well as the different clones present in the sample. **B)** Alignment of reads to the *BCR-ABL1* reference sequence. The grey area shows reads for a CML sample (patient P3,49 months) produced from one SMRT cell on the PacBio RSII instrument. The sequencing generates a uniform coverage of about 10,000X over the entire reference sequence. The red vertical line indicates the presence of a T315I mutation, present in 88.9% of the reads. The mutation F359C was also detected in this sample at a frequency of 4.2% and can be seen as a faint vertical line. **C)** Results of a dilution experiment of the CML sample in panel B) (P3, 49 m). The leftmost bars show mutation rates of T315I (red) and F359C (blue) for the undiluted sample. To the right are observed mutation frequencies for a dilution series where the expected T315I frequency reached 50%, 10%, 1% and 0.5%. The expected frequencies of T315I and F359C are shown in red and blue letters, respectively. Positions marked with ‘X’ indicate mutations not detected by the PacBio sequencing.

We designed a simple workflow with a single step PCR-amplification aimed comprising a 1,6 kb fragment of the fusion transcript and excluding the wild-type *ABL1*. This fragment was then sequenced using the PacBio sequencing protocol. We estimate that the whole process from cDNA synthesis to PacBio sequencing and analysis can be performed within 2–3 days (see Figure 1A). Results from one CML patient (patient 3) show that sequencing on a single PacBio SMRT cell generated a uniform mapped read coverage of about 10,000X across the entire length of the *BCR-ABL1* amplicon (Figure 1B). In order to evaluate the sensitivity and specificity of our assay, serial dilutions from patient P3 harboring T315I and F359C *ABL1* mutations were diluted into wild type *BCR-ABL1* and analyzed. Our results show that the T315I mutation could be detected down to an expected frequency of 1%, while F359C was found down to 0.5% (Figure 1C). This is in concordance with recent MPS studies that detected mutations down to 1% [4,8]. Importantly, not a single mutation other than T315I and F359C were found in any of the 5 samples in the dilution series, indicating a 0% false positive rate and thus a perfect specificity for these samples. The low false positive rate is likely explained by the random distribution of sequencing errors inherent to the PacBio technology [10,11], which results in highly accurate base calls for molecules that are sequenced at high coverage.

**Figure 2:**
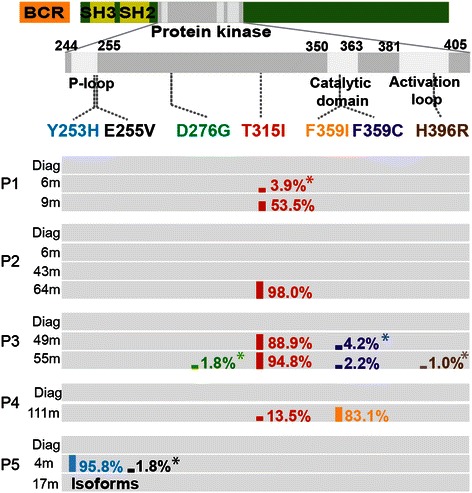
***BCR-ABL1*mutations and their composition in patient samples.** Overview of *BCR-ABL1* mutations detected in five CML patients (P1-P5) at the time of diagnosis and at subsequent follow-up examinations following TKI treatment. Samples taken at the time of diagnosis are labeled ‘Diag’. The follow-up samples are labeled with the number of months after diagnosis. The numbers next to the colored bars show the frequencies of all mutations observed by PacBio sequencing. Asterisks (*) indicate mutations that failed to be detected by Sanger sequencing.

We further analyzed samples taken at the time of diagnosis as well as at later time points following TKI treatment for six patients with limited or no molecular response to TKI treatment (see Table 1). All together, 22 samples were sequenced generating an average of 32,000 CCS reads per sample (Table 1). We identified a total of 13 mutations distributed over the residues Y253H, E255V, D276G, T315I, F359I, F359C and H396R (Figure 2). All mutations have previously been implicated in resistance to one or more TKIs. Besides, all of these positions, except the D276, are among the 12 key positions previously reported in compound mutants [5]. In all six patients, the PacBio system successfully confirmed all mutations previously detected by Sanger sequencing. Moreover, we identified 5 mutations present at low frequency (below 5%) that previously failed detection with Sanger sequencing. Two of the patients were carrying a single mutation, three patients carried more than one mutation and for the last patient no point mutations were detected. The results for the individual patients are described in more detail below.

### Patients with the T315I as a single mutation

**Figure 3:**
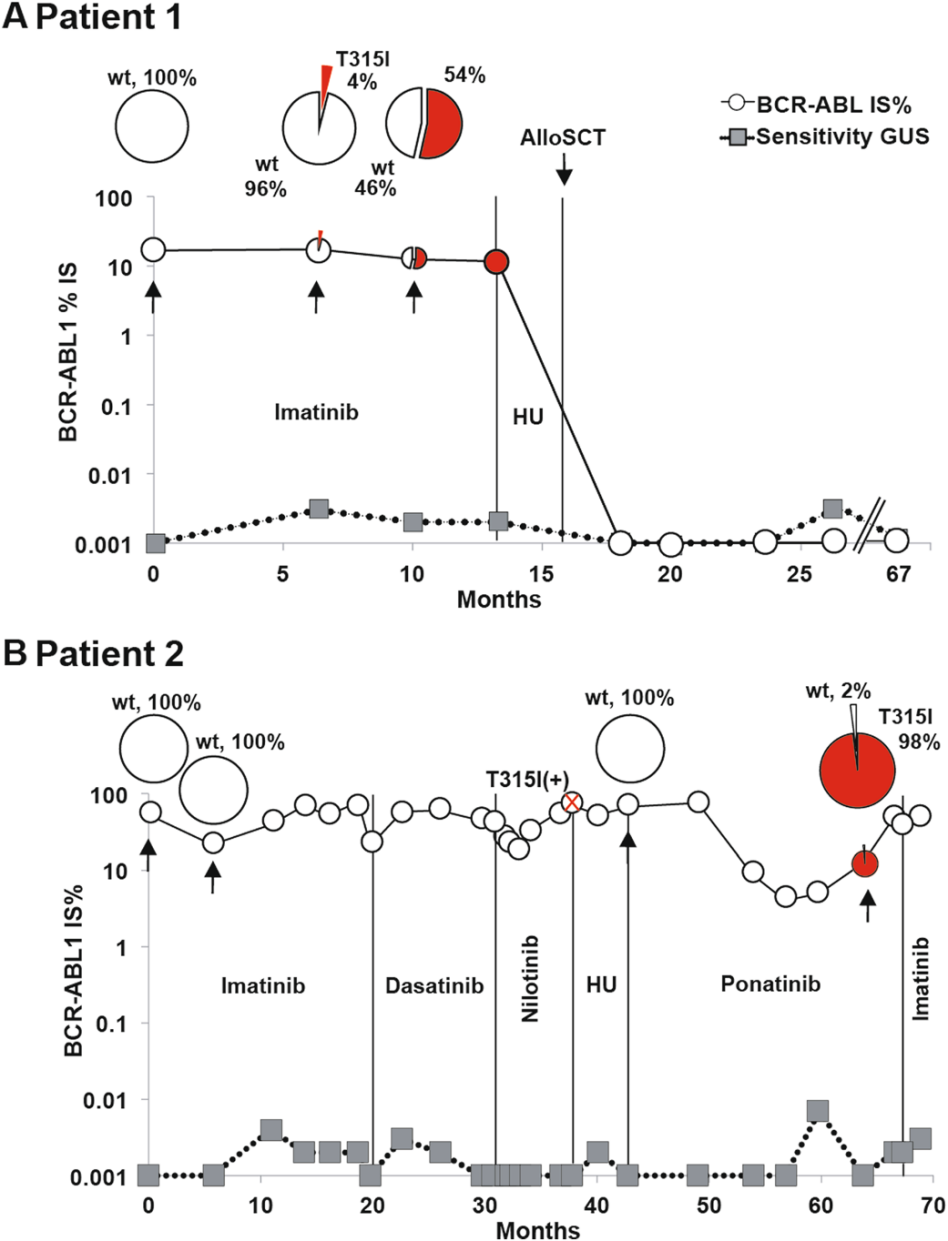
**Overview of treatments and PacBio results for patients with single mutations. A)** Results for patient 1. The *BCR-ABL1* IS% values measured by routine quantitative RT-PCR are shown in open circles. The sensitivity of this assay was measured for the *BGUS* reference gene and depicted by gray squares. The samples that were analyzed by PacBio sequencing are indicated by black arrows and their mutation composition showed in the circle plot diagrams above each time point. Vertical lines indicate the treatment periods. HU (Hydroxyurea). **B)** Results for patient 2. The T315I mutation was detected after nilotinib treatment, as indicated by the red cross. The mutation was detected at this time point using our allele specific quantitative PCR used in routine analysis.

We detected only the T315I mutation in two patients (1 and 2) (Figure 3). As shown in Figure 3A, patient 1 was diagnosed with chronic phase CP-CML. No significant molecular remission was seen after 15 months of imatinib therapy and the patient underwent allogeneic stem cell transplantation (SCT). At present the patient is in complete molecular remission. It is noteworthy that, in this case the T315I was detected with Sanger sequencing 13 months after diagnosis while a small clone (4%) could be detected in a sample taken four months earlier using the PacBio sequencing.

Patient 2 shown in Figure 3B was diagnosed with CP-CML with no significant molecular response following imatinib therapy. After 18 months, the patient received dasatinib followed by switch to nilotinib after four months, and then to hydroxyurea after further three months. The patient progressed to AP-CML during hydroxyurea treatment and at 38 months after diagnosis the T315I mutation could be detected by routine analysis using allele specific qPCR and Sanger sequencing. Notably, at this point a Philadelphia positive clone with del (11q) was found in the karyotype analysis. After few months on hydroxyurea the patient lost the T315I mutated clone and the PacBio analysis did not detect the T315I or any other mutation (Figure 3B). These results were in accordance with the Sanger sequencing and qPCR analysis. The patient then received ponatinib treatment, outside the PACE study. After an initial molecular response and following twelve months of ponatinib therapy, molecular relapse occurred. As measured by the PacBio sequencing, the T315I mutation reached 98% at this point but no other mutations were detected (Figure 3B). We can thus speculate that in this patient *BCR-ABL1*-independent factors explain TKI resistance. The patient was switched back to imatinib therapy due to severe cardiovascular side effects from ponatinib and rising T315I values. The latest karyotype analysis showed Philadelphia positive cells only, with all cells containing the previously found del (11q).

### Patients carrying several mutations

**Figure 4:**
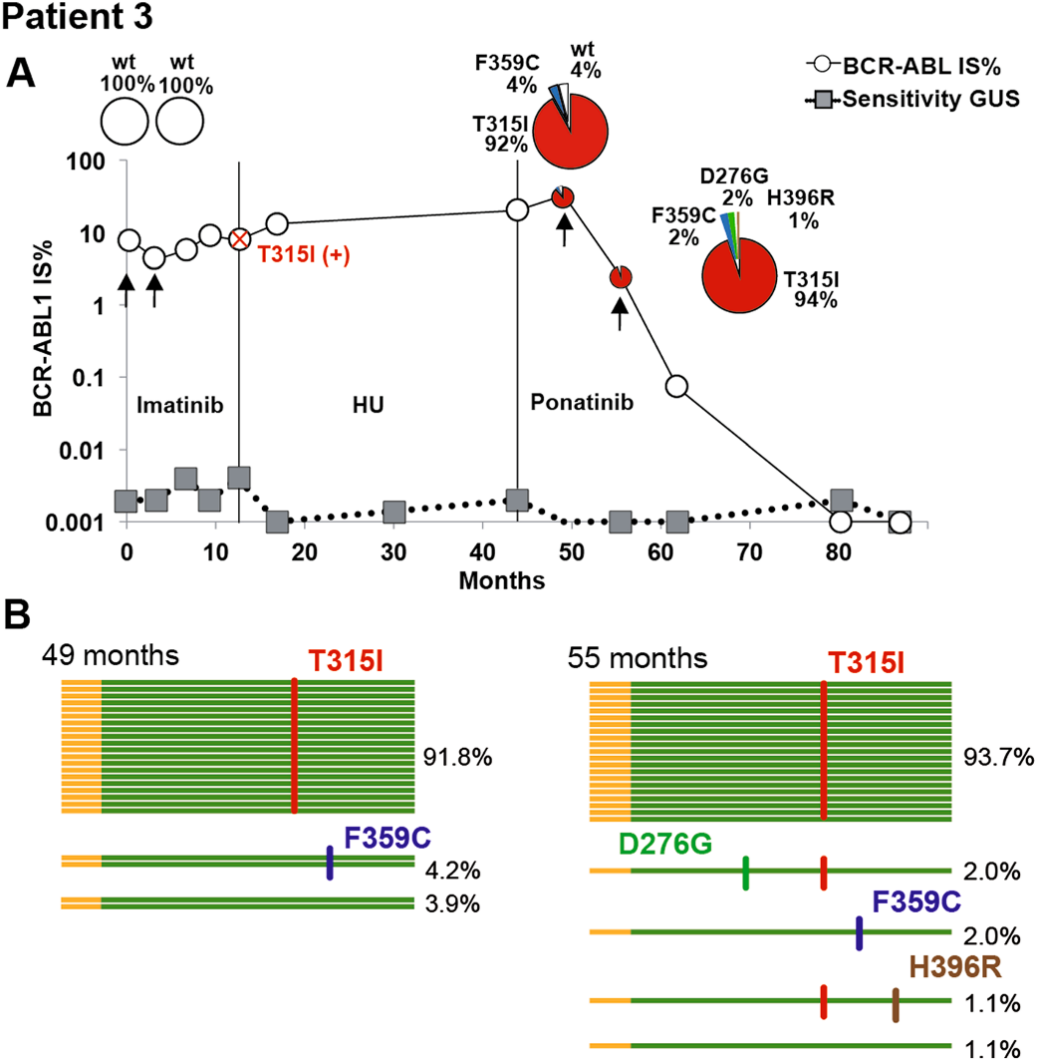
**Overview of treatments and PacBio results for patient 3. A**) The *BCR-ABL1* IS% values measured by routine quantitative RT-PCR are shown in open circles. The sensitivity of this assay was measured for the *BGUS* reference gene and depicted by gray squares. As indicated by the red cross, the T315I mutation was detected after eleven months of imatinib treatment. The mutation was detected at this time point using our allele specific quantitative PCR used in routine analysis. The samples that were analyzed by PacBio sequencing are indicated by black arrows and their mutation composition showed in the circle plot diagrams. Vertical lines indicate the treatment periods. HU (Hydroxyurea). **B)** This panel shows the mutational composition in the *BCR-ABL1* transcript for the 49 m and 55 m follow-up samples. Horizontal lines gives a schematic representation of high-quality PacBio reads that were used for examining the mutational composition. At 49 m, 91.8% of the reads carried T315I mutation. 4.2% of the reads showed the presence of F359C and 3.9% of the reads contained none of the mutations. At 55 m, two new clones appeared, one containing D276G and T315I (2.0% of the reads) and one containing T315I and H396R (1.1% of the reads).

More than one mutation was present in three of the patients (3,4 and 5) (Figure 2). As shown in Figure 4A Patient 3 was diagnosed with CP-CML with no significant molecular response to imatinib. Due to the occurrence of T315I mutation the therapy was switched to hydroxyurea to avoid further selection of the T315I positive clone. The patient progressed to accelerated phase (AP) after 9 months of hydroxyurea therapy. The patient was then included in the PACE clinical trial. After a few months of ponatinib treatment the T315I mutation was still detected and with the occurrence of another mutation F359C. The PacBio sequence analysis showed that the mutations were localized in different molecules (see Figure 4B). After additional months of treatment two small T315I positive clones emerged, carrying the D276G (2%) and the H396R (1%) respectively, while the F359C positive clone was reduced by half. Interestingly, a Philadelphia negative cell clone harboring del (5q) in the karyotype emerged 6 months after ponatinib study inclusion and has over time become the prevalent clone in this patient.

**Figure 5:**
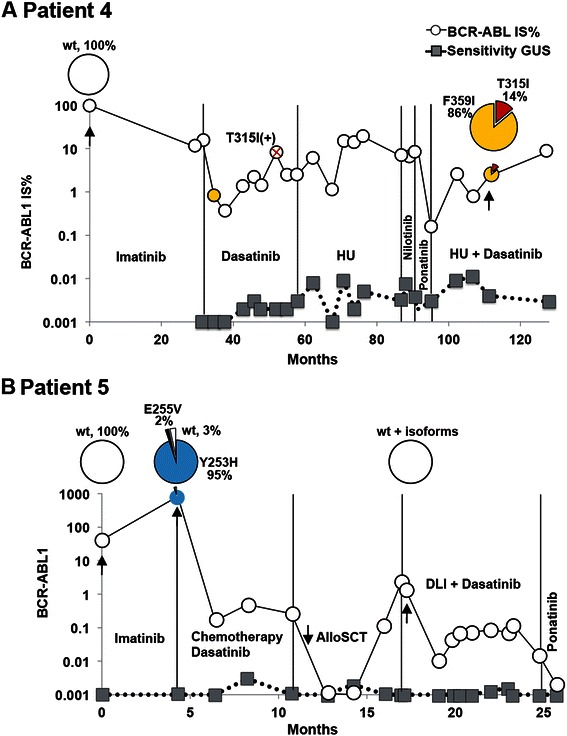
**Overview of treatments and PacBio results for patients 4 and 5. A)** Results for patient 4. The *BCR-ABL1* IS% values measured by routine quantitative RT-PCR are shown in open circles. The sensitivity of this assay was measured for the *BGUS* reference gene and depicted by gray squares. As indicated by the red cross, the T315I mutation was detected after 58 months of dasatinib treatment. The mutation was detected at this time point using our allele specific quantitative PCR used in routine analysis. The samples that were analyzed by PacBio sequencing are indicated by black arrows and their mutation composition showed in the circle plot diagrams. Vertical lines indicate the treatment periods. HU (Hydroxyurea). **B)** Results for patient 5. Measurements shown were made as in A. AlloSCT: allogeneic stem cell transplantation. DLI: donor lymphocyte infusion.

Patient 4 (Figure 5A) was diagnosed with CP-CML and initially treated with hydroxyurea and interferon followed by imatinib therapy for 36 months with minor molecular response, due to the F359I and F359V mutations detected at this point by Sanger sequencing. Therapy change to dasatinib resulted in a molecular response, however followed by a later increase in *BCR-ABL1* transcripts and emergence of the T315I mutation. The treatment was changed to hydroxyurea plus dasatinib, followed by two short periods with nilotinib and ponatinib (PACE study) treatments respectively. Due to severe side effects of ponatinib, the patient was switched back to the previously used combination of hydroxurea and dasatinib. At present the patient shows only minor molecular response. The PacBio analysis was performed during this last treatment period and showed the F359I (86%) and the T315I (14%) mutations in separate clones. Further analysis is required to evaluate the future clonal evolution and treatment response to the present therapy.

Patient 5 was diagnosed in late CP-CML. Therapy changed from imatinib to nilotinib four months post diagnosis (Figure 5B). After one month on nilotinib therapy, the patient developed Y253H (95%) and E255V (2%) mutations and went into a blast crisis. The patient then received chemotherapy (following the “EWALL” protocol) and dasatinib. Allogeneic SCT was performed but four months post SCT the patient again presented increasing *BCR-ABL1* values. Interestingly at this point the patient showed none of the previously seen point mutations but instead we could detect *BCR-ABL1* transcript isoforms. The patient then received donor lymphocyte infusion and dasatinib to later go under ponatinib treatment. The patient is at present in MR 4,5 continuing the ponatinib therapy. According to the sequencing results these two mutations resided in separate molecules. The Y253 and E255V mutations are well known P-loop mutations causing resistance to imatinib and nilotinib while sensitive to dasatinib when either found individually or in the same molecule [5]. However, the compound mutant shows resistance to ponatinib, while the two mutants are individually sensitive.

### Patients with transcripts isoforms

**Figure 6:**
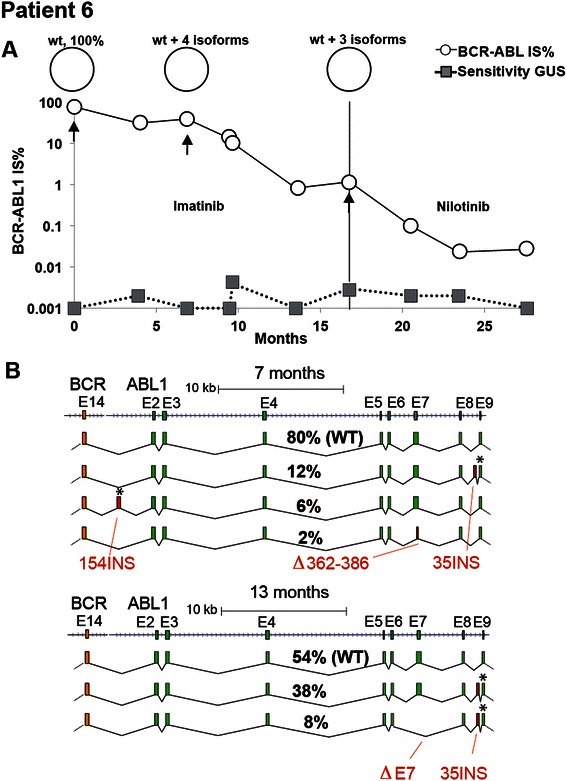
**Overview of treatments and PacBio results for patient 6. A)***BCR-ABL1* IS% values measured by routine quantitative RT-PCR are shown in open circles. The sensitivity of this assay was measured for the *BGUS* reference gene and depicted by gray squares. The samples that were analyzed by PacBio sequencing are indicated by black arrows. The vertical line indicates the treatment period. **B)** This panel shows *BCR-ABL1* isoforms in patient 6. At 7 months post diagnosis four different splice isoforms were identified. The most common isoform was the ‘wild type’ (WT) *BCR-ABL1* transcript isoform, i.e. identical to the reference sequence used for mapping, present in 80% of the molecules. Two other isoforms contained insertions of entire exons, of lengths 35 bp and 154 bp, respectively and one contained a partial deletion of exon 7 of *ABL1*. At 13 months post diagnosis the WT isoform was present in 54% of the molecules whereas isoforms containing the 35 bp insertion between exon 8 and 9 in *ABL1* was present in the other two isoforms.

Besides establishing the clonality of mutations, our method could also identify transcript isoforms in patients 5 and 6. As discussed above, patient 5 developed transcript isoforms when losing molecular response after allogeneic stem cell transplantation (Figure 5). Patient 6 was diagnosed with CP-KML and showed slow molecular response after 18 months receiving imatinib (Figure 6A). The patient responded better to nilotinib therapy and at present the patient has reached MMR. In this case several splice isoforms were present both at 7 and 13 months post diagnosis (Figure 6B). No less than four different isoforms were identified in the sample taken 7 months after diagnosis with two lower frequency isoforms detected besides the WT transcript and 35INS. One isoform had a 154 bp insertion between exon 14 of *BCR* and exon 2 of *ABL1*, leading to a truncated protein (present in 6% of transcripts), while the other isoform had a deletion of 24 amino acids in exon 7 of *ABL1* (present in 2% of transcripts). Notably, the frequency of the wild type *BCR-ABL1* wild type isoform decreased from 80% to 54% between the two time points (6C), while splice isoforms carrying an insertion of an additional 35 bases (35INS) between exons 8 and 9 in *ABL1* increased in frequency. This exact 35 bp insertion has been reported previously in patients undergoing kinase inhibitor therapy [12].

## Discussion

Our results show that mutations can be detected at a level of ~1% in a background of wild type *BCR-ABL1* making it a useful tool for screening of both high and low level kinase domain mutations. Further, it provides information on the clonal distribution of mutations as well as *BCR-ABL1* isoforms in a single assay. This feature is of major clinical relevance as compound mutations show different resistance profiles compared to individual mutants [5]. Standard Sanger sequencing methods routinely used in diagnostic laboratories are unable to distinguish between independent or compound mutations. Until now, this information has only been available through time consuming cloning experiments [5], underlining the potential clinical utility of our assay.

Although recent reports showing MPS-approaches are emerging [8,9], its usefulness in establishing the clonality of mutations has recently been debated. In a recent report, Parker et al. [13] showed that compound mutations detected by MPS technologies might actually be artifacts due to PCR-mediated recombination. However, our assay has a somewhat different setup compared to previous studies. Instead of performing a two round nested PCR, as is required for shorter read technologies, the fusion transcripts were amplified in one single round. This could potentially reduce the rate of PCR recombination. We were able to evaluate the degree of *in vitro* artifacts in our experiments. For example, in the 49-month sample from patient 3, 91.2% of the reads contained only T315I and 4.2% of the reads contained only F359C (see Figure 4B). In the same sample 0.1% of reads show the presence of both T315I and F359C. These results suggest that the recombination rate in this particular case is very low, well below the frequency of the individual clones. However, since the rate of chimeric reads can be influenced by the experimental conditions and may vary between samples, a more thorough investigation would be required to validate the PCR recombination rate under different circumstances.

On the practical level, the PacBio assay allows for a simple, efficient and streamlined workflow conducive to clinical routine. Our laboratory workflow provides a quick turnaround time of approximately two days, encompassing all steps from RNA isolation to report generation. A simple library preparation procedure, rapid sequencing and straight forward bioinformatics analysis enable this efficient workflow. The library preparation is performed during one day and the sequencing run takes approximately 2–3 hours per sample. Under the current set up, PacBio sequencing is more expensive compared to the more traditional Sanger sequencing and RQ-PCR based assays. However, the cost for sequencing of small target regions such as the *BCR-ABL1* transcript is comparable to that of other available MPS technologies. In the present study, we obtained 10,000X coverage of *BCR-ABL1* for each of the samples. In light of this rather extensive coverage, it is likely that a similar sensitivity for mutation detection could to be obtained when utilizing a reduced coverage, thus opening up the possibility of barcoding of two or more samples on one SMRT cell. Further, due to the continuous improvements of the PacBio system in terms of quality, read length and throughput, the potential for multiplexing is likely to increase, thus leading to substantial reductions in experimental cost.

This study presents a proof of principle for detection of *BCR-ABL1* mutations and our results are based on just a handful of patients, limiting the generality of our conclusions. Nevertheless the analysis of individual patient samples illustrates important aspects and strengths of our approach. One main advantage is the sensitivity of the assay, as illustrated in one of the patients (patient 1) where we could detect the T315I mutation four months earlier than detected by Sanger sequencing. These results indicate that an NGS-screen could be informative when performed at earlier time points, possibly in patients with no or limited responses to TKI therapy already at the three months control. Further studies are needed to specifically address this question.

The sensitivity of the method can also be instrumental in excluding other *BCR-ABL1* mutations as responsible factors for the observed TKI resistance. For example, in patient 2, we could only detect the T315I and despite an initial molecular response to ponatinib, the patient remains with a minor molecular response. Thus in this case *BCR-ABL1*-independent factors might explain the failed therapy. This information is of particular importance when looking for alternative TKI-resistance pathways.

The ability to discern between independent and compound mutations is a major advantage of this assay. For example, patient 3 carried both the T315I and F359C mutations, but present in independent clones. A recent study has shown that the compound mutation F359C and T315I is associated with in vitro profiles implicating mutant pairing of these two positions in moderate and high-level resistance to ponatinib and rebastinib, respectively [5], while the individual mutants are instead sensitive to these substances. Thus, we can speculate that the molecular response observed in this case upon ponatinib treatment, can be explained by the fact that these mutations are located in different molecules in this patient. In contrast, the T315I positive clone (84%) acquired independently two extra mutations (D276G and H396R) upon ponatinib treatment. These low frequency compound sub clones did not seem to impede the molecular response (MR5) attained after 3 years of treatment. The results are somehow conflicting with recent results showing that the compound mutant H396R/T315I has an intermediate to high resistance *in vitro* profile with an IC_50_ for ponatinib of 90.8 ± 24.7 nM compared to an IC_50_ of 20.1 ± 3.5 (H396R) and 29.1 ± 7.8 nM (T315I) for the individual mutants [5]. However, *in vitro* sensitivity does not necessarily always correlate with the clinical response to treatment.

Similarly, patient 5, harboring the non-compound Y253H/E255V mutations, would clearly benefit today from sensitive examination of the clonal composition of these two mutations prior to therapy decisions because of the high ponatinib-resistance of the Y253H/E255V compound mutant [5]. The fact that in this patient the mutations occur in different molecules would remain indiscernible by routine Sanger sequencing.

Another advantage of our approach is that as we amplify and sequence almost the entire *BCR-ABL1* p210 transcript we are able to identify transcript isoforms. We identified elevated levels of transcripts isoforms in two of the patients. Among the variants that we detected, 35INS is best studied in the literature. Some studies have described 35INS as a possible mechanism of imatinib resistance [14,15], while biochemical data from a separate study shows that it does not contribute to TKI resistance in vitro [16]. Although there is at present no basis for taking 35INS into consideration for treatment decisions, these conflicting reports highlight the need for routine screening in CML patients in order to gain more knowledge. Our results suggest that the PacBio assay can be used for screening of 35INS as well as other splice isoforms down to a frequency of at least 5%. Furthermore, it enables simultaneous detection of multiple different alternative isoforms present in a single sample. These results corroborate previous findings that propose alternative splicing as a common mechanism among CML patients undergoing TKI treatment [14,17]. However, to clarify the role of transcript isoforms in drug resistance and response a larger number of samples should have to be analyzed and our approach might simplify this kind of analysis.

Although this study is focused on *BCR-ABL1* the same method can be readily applied to the analysis of other cancer-associated fusion transcripts, providing not only information on the clonal distribution of mutations but also on isoform frequencies. Isoform analysis is to this date not performed routinely on CML patient samples and therefore the knowledge on their impact on disease progression and treatment efficiency is very limited.

## Conclusions

This study presents an NGS method for the detection of *BCR-ABL1* mutations associated with TKI-resistance. We show a simple workflow with a rapid turn around time. The strengths of this method are its sensitivity, enabling early detection of resistance mutations, and its capability to discern between individual and compound mutations. Thus we believe that this assay can potentially be introduced into clinical practice to guide therapeutic decisions for TKI resistant patients, provided thorough validation in a larger number of samples.
